# Spiral Steel Wire Based Fiber-Shaped Stretchable and Tailorable Triboelectric Nanogenerator for Wearable Power Source and Active Gesture Sensor

**DOI:** 10.1007/s40820-019-0271-3

**Published:** 2019-05-11

**Authors:** Lingjie Xie, Xiaoping Chen, Zhen Wen, Yanqin Yang, Jihong Shi, Chen Chen, Mingfa Peng, Yina Liu, Xuhui Sun

**Affiliations:** 1 Institute of Functional Nano and Soft Materials (FUNSOM), Jiangsu Key Laboratory for Carbon-Based Functional Materials and Devices, Soochow University, Suzhou, 215123 People’s Republic of China; 2 Nantong Textile and Silk Industrial Technology Research Institute, Jiangsu Industrial Technology Research Institute of Textile and Silk, Nantong, 226314 People’s Republic of China; 3 Department of Mathematical Sciences, Xi’an Jiaotong-Liverpool University, Suzhou, 215123 People’s Republic of China

**Keywords:** Triboelectric nanogenerator, Stretchable, Human motion energy, Wearable power source, Active gesture sensor

## Abstract

Continuous deforming always leads to the performance degradation of a flexible triboelectric nanogenerator due to the Young’s modulus mismatch of different functional layers. In this work, we fabricated a fiber-shaped stretchable and tailorable triboelectric nanogenerator (FST–TENG) based on the geometric construction of a steel wire as electrode and ingenious selection of silicone rubber as triboelectric layer. Owing to the great robustness and continuous conductivity, the FST–TENGs demonstrate high stability, stretchability, and even tailorability. For a single device with ~ 6 cm in length and ~ 3 mm in diameter, the open-circuit voltage of ~ 59.7 V, transferred charge of ~ 23.7 nC, short-circuit current of ~ 2.67 μA and average power of ~ 2.13 μW can be obtained at 2.5 Hz. By knitting several FST–TENGs to be a fabric or a bracelet, it enables to harvest human motion energy and then to drive a wearable electronic device. Finally, it can also be woven on dorsum of glove to monitor the movements of gesture, which can recognize every single finger, different bending angle, and numbers of bent finger by analyzing voltage signals.

**Electronic supplementary material:**

The online version of this article (10.1007/s40820-019-0271-3) contains supplementary material, which is available to authorized users.

## Highlights

- Owing to the great robustness, continuous conductivity, and geometric construction of a steel wire electrode, the FST–TENGs demonstrate high stability, stretchability, and even tailorability.
- By knitting several FST–TENGs to be a fabric or a bracelet worn on the human body, it enables to harvest human motion energy.
- The FST–TENGs can also be woven on dorsum of glove to monitor the movements of gesture.

**Electronic supplementary material:**

The online version of this article (10.1007/s40820-019-0271-3) contains supplementary material, which is available to authorized users.

## Graphical abstract

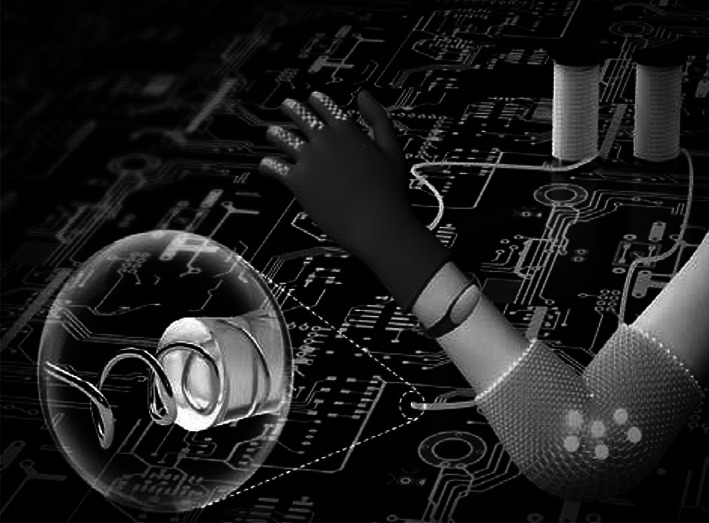

## Introduction

The progress in human’s life has benefited from the rapid development of multipurpose wearable electronics, such as smart watches, smart glasses, electronic skins, etc. [1, 2]. These electronics could mimic the characteristics of human skin and wireless sensing networks that monitor human health and motion track [3–5]. Several challenges have been raised for wearable devices that are desired to be flexible, lightweight, low price, and still stable [6, 7]. Nevertheless, commercialized portable energy storage units including batteries and supercapacitors are relatively heavy, charged frequently, and face critical lifetime limitation [8–10]. To overcome these challenges, a series of advanced energy-harvesting technologies based on triboelectric, photovoltaic, and thermoelectric effects from ambient environment for sustainable and portable power source have been developed [11–15].

Compared with solar energy and thermal energy, mechanical energy harvesting is whenever and wherever possible that is independent of weather and environment. Triboelectric nanogenerator (TENG), based on coupling effects of triboelectrification and electrostatic induction, has been one of the most effective strategies to convert various types of mechanical energies into electricity, such as human motions, wind, water wave, rain drops, and vibrations [16–21]. It has achieved rapid progress as a sustainable power source with advantages of low-cost, high output, lightweight, and wide choice of materials for the usage in extensive devices [22–26]. Attempts have been made to fabricate flexible TENGs for potential applications in wearable power sources [27–31]. The ability to withstand complex mechanical deformation is limited owing to the Young’s modulus mismatch of interface compatibility of triboelectric layer and electrode layer. Recently, there have been three general strategies to make stretchable conductors to match stretchable triboelectric materials such as polydimethylsiloxane (PDMS) or silicone rubber [32]: including deterministic geometries of rigid materials to elongate, dispersing conductive particles in elastomer, and utilizing conductive materials that are intrinsically stretchable [28, 33–35]. However, for the stretchable fiber-based power textiles, it is needed to be realized by designing reasonable geometrical shape.

In this work, we present and tailorable (FST–TENG) to harvest human motion energy for wearable electronics. The spiral steel wire is selected as the electrodes, which is highly stretchable due to its special structure, while silicone rubber is employed to cover the spiral steel wire as the triboelectric layer. The geometric design enables every single FST–TENG to be stretchable and relatively stable within the range of ~ 50% stretch. In addition, fabrics and bracelet that worn on the body or wrists knitted with several FST–TENG fibers have been fabricated. To evaluate the performance of the FST–TENG fabric on power generation, the FST–TENG fabric is used to light up LEDs, charge commercial capacitors, and then drive an electronic watch for demonstration. Finally, the FST–TENG can also be applied for inspecting each single finger, different bending degree and identifying digital gestures by the smart glove with FST–TENGs attaching.

## Experimental Section

### Materials

Spiral steel wire is 0.3 mm of wire diameter (d), 2 mm of the outer diameter (D), 1.7 mm of medium diameter (D_2_), 0.618 mm of pitch (t), and 8° of spiral angle (α), which is purchased. Silicone rubber (Ecoflex 00-30) was purchased from Smooth-On, Inc. and used as-received.

### Fabrication of the FST–TENG

The curing agent and liquid silicone rubber (1:50 volume ratio) were mixed in a beaker. The mixture was mixed uniformly and then injected in the two half-circular acrylic molds. A spiral steel wire was inserted in the mixture. After combining these two half-circular tubes for encapsulation, the silicone rubber is curing naturally at room temperature. Finally, the cured silicone rubber was demolded from the acrylic tubes to get the FST–TENG and weave multiple FST–TENG into cloth and bracelet.

### Characterization and Measurements

An optical microscope (DM4000M) was used to investigate the morphology and interior structure of the FST–TENG. The sheet resistances (*R*_s_) of the spiral steel wire electrodes were measured by using the four-point van der Pauw method with collinear probes (0.5 cm spacing) connected to a Keithley 2400 Sourcemeter. The Electromechanical Universal Testing Machine (E44 104) was used to test the mechanical properties of the spiral steel wire and FST–TENG. For the electrical output measurement of the FST–TENG, an external tapping force was applied by a commercial linear mechanical motor (Winnemotor, WMUC512075-06-X) with hogskin to simulate the skin touching and a programmable electrometer (Keithley model 6514) was used to test the *V*_oc_, *I*_sc_, and *Q*_sc_. During the simulation process of linear motor, the maximum distance between the FST–TENG and hogskin was 4 cm. Electronic universal testing machine (Instron 3365) was used to test tensile properties of the spiral steel wire.

## Results and Discussion

**Fig. 1:**
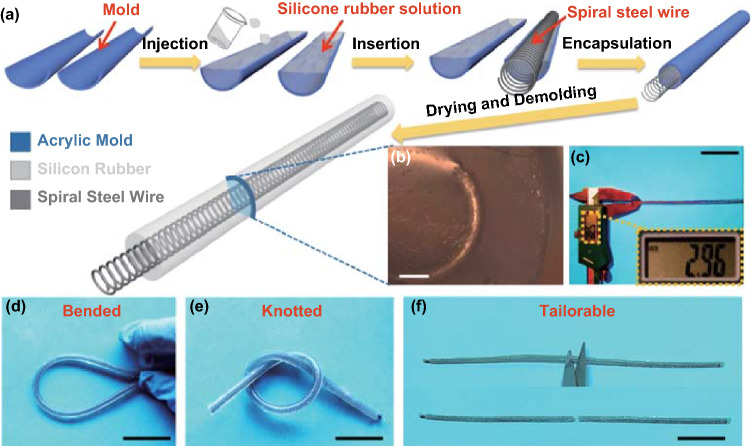
Schematic illustration and mechanical behaviors of the spiral steel wire electrode based fiber-shaped stretchable and tailorable triboelectric nanogenerator (FST–TENG). **a** Fabrication process of FST–TENG. **b** Cross-sectional optical microscope image (scale bar: 500 μm). **c** Photograph of the diameter measurement of FST–TENG (scale bar: 2 cm). Photograph of the FST–TENG at different states: **d** bended, **e** knotted, and **f** tailorable state (scale bars: 3 cm)

The schematic illustration and mechanical behaviors of the FST–TENG are schematically illustrated in Fig. 1. Here, silicone rubber was chosen as the triboelectric and packaging materials of the FST–TENG due to excellent softness, toughness, stretchability, and strong tendency to gain electrons. A commercial steel wire that designed to be spiral shape was employed as the electrostatic electrode. For the fabrication of a FST–TENG, an acrylic tube was cut into two half-pipes as molds. Silicone rubber was coated evenly on the spiral steel wire and finally a fiber with controlled diameter can be obtained. After encapsulating the two molds and drying silicone rubber, the fiber demolded from the molds to get the final device (Fig. 1a). The detail fabrication process can be found in the Experimental Section. From the cross-section optical microscope image of the FST–TENG, it can be observed that a spiral steel wire with the outer diameter (*D*) and wire diameter (*d*) of ~ 2 mm and ~ 200 μm, respectively, is located in the central part of the silicone rubber (Fig. 1b). Moreover, a typical FST–TENG with the diameter of ~ 2.94 mm (Fig. 1c) can be not only bended (Fig. 1d) and knotted (Fig. 1e) but also tailorable (Fig. 1f), respectively. Since the silicone rubber and the spiral steel wire electrodes are terrific stable, for practical application, the FST–TENG can be tailored into arbitrary separate devices. All tailorable energy devices can still be used to collect the mechanical energy of the different parts of the human body [36, 37].

**Fig. 2:**
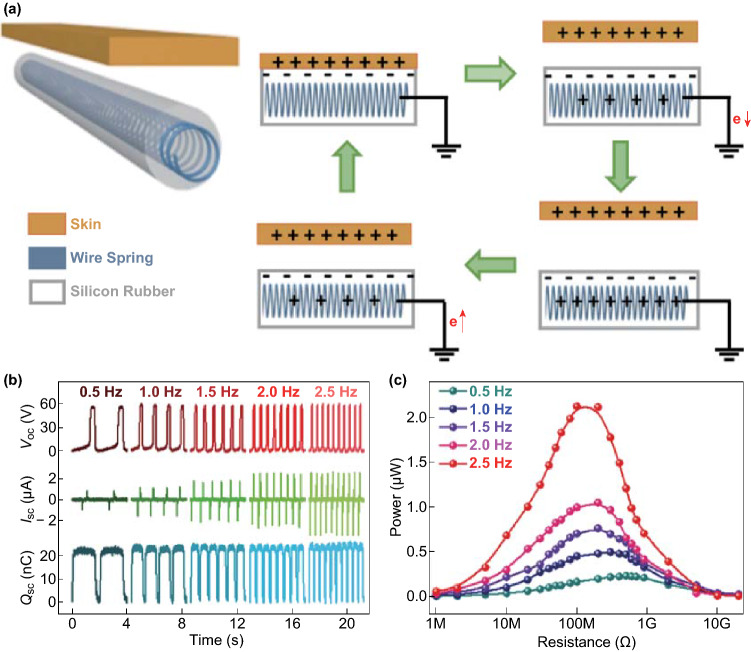
Working mechanism and electrical output performance of FST-TENG at single-electrode mode. **a** Schematic illustration of working mechanism for generating electricity under short-circuit conditions. **b** Electrical outputs of the spiral steel wire based TENG with different motion frequencies ranging from 0.5 to 2.5 Hz, including *V*_oc_, *I*_sc_, and *Q*_sc_. **c** Dependence of the output average power under external load with different frequencies from 0.5 to 2.5 Hz

The working mechanism and electrical output performances of the FST–TENG are shown in Fig. 2. The spiral steel wire is connected to the ground and skin is acted as another triboelectric material for generating electricity. The single-electrode mode FST–TENG is based on the coupling effect of triboelectrification and electrostatic induction [38], as schematically illustrated in Fig. 2a. In initial state, when skin contacts with the silicon rubber, the negative triboelectric charges would maintain on the silicone rubber surface and the generated positive charges on the skin (State I). When the skin detaches, the electrons will flow from the conductive steel wire to the ground under the short-circuit condition and the positive charges will be induced in the electrode (electrostatic induction effect) (State II). Until skin is quite far away from silicone rubber surface, the transferred charges from the spiral steel wire electrode to the ground will reach their maximum values (State III). Then the attaching skin causes the electrons to flow back in the reverse direction due to reverse electrostatic induction (State IV). When skin goes back to its original position, the charged surface comes into full contact again, while the triboelectric charge distribution of the FST–TENG returns to its original state. Through continuous circulation of the contact and separate process between the skin and FST–TENG, reciprocating motion of electrons between the spiral steel wire electrode and the ground can cause an alternating current and power output. To characterize the FST–TENG, the output performance was evaluated by a cyclic movement through a linear motor. Through mutual contacting and separation of different triboelectric materials with FST–TENG in single-electrode mode, corresponding transferred short-circuit charge (*Q*_sc_), open-circuit voltage (*V*_oc_), and short-circuit current (*I*_sc_) of these materials which reflect the triboelectric ability to lose electrons were recorded (Fig. S1). Compared with Cu, Al, and nylon with same area of 6 × 6 cm^2^, hog skin which simulates the human skin has the more electrostatic charges generated at the contacting interface and can also effectively improve the contact area due to its certain flexibility, and thus has the higher output *Q*_sc_ [39]. Figure 2b exhibits the results of *V*_oc_*, I*_sc_ and *Q*_sc_ for the FST–TENG under the motion frequencies ranging from 0.5 to 2.5 Hz, respectively. It can be seen that in Fig. 2c, with the increase of the motion frequencies, the *V*_oc_ (~ 59.7 V) and *Q*_sc_ (~ 23.7 nC) keep almost constant, while the peak value of the *I*_sc_ increases from 0.84 to 2.67 μA with the motion frequency. The average power of the FST–TENG measured at different external load resistances increases with the motion frequency. The maximum power value (~ 2.13 μW) can be achieved at the load resistance of 100 MΩ at 2.5 Hz. It is observed that the optimum resistance decreases with the increasing frequency, which can be explained by the decrease of the impedance of TENG with the increase of the motion frequency [20]. Also, for long-term application, the stability has been tested by contacting and separating the FST–TEMG for 5000 times, as illustrated in Fig. S2a. It is easy to see that the *V*_oc_ during the first 50 cycles is close to the last 50 cycles, which verifies excellent stability and reliability. The durability of the FST–TENG was examined under circulating between 180° bended and 50% stretching strain motion for 5000 cycles by the linear motor, as shown in Fig. S2b. Normalized *V*_oc_, *I*_sc_, and *Q*_sc_ values of FST–TENG were recorded after every 500 times of cycling, which shows no significant degradation in performance, confirming their excellent flexibility, stretchability, and stability. To investigate the tailorability of the FST–TENG, an 8 cm long fiber was cut into two parts of equal length, i.e., 4 cm. The *V*_oc_ (~ 49.5 V), *I*_sc_ (~ 1.01 μA), and *Q*_sc_ (~ 17.3 nC) of both parts are almost half of the electric output of the original 8 cm long one, revealing that the tailoring process hardly affects the performance of the FST–TENG, as shown in Fig. S3.

**Fig. 3:**
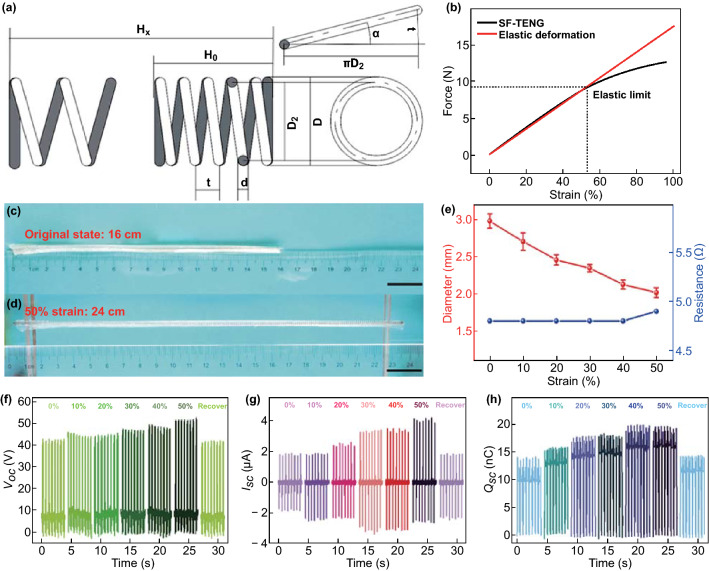
Output performance of the FST–TENG under different strain level. **a** Geometrical diagram of stretching spiral steel wire and **b** stress–strain curve of FST–TENG. The photograph of the spiral steel wire-based TENG **c** at original state and **d** stretched state (scale bar: 2 cm). **e** Dependence of the resistance and diameter of the spiral steel wire-based TENG under different strain levels (0–50%). Electrical output of the FST–TENG under various strain levels including **f** *V*_oc_, **g** *I*_sc_, and **h** *Q*_sc_

The general performance of the stretchable FST–TENG under different strain level is schematically displayed in Fig. 3. The Young’s modulus of steel wire (2 × 10^11^ N m^−2^) is much higher than that of silicone rubber (1.2 × 10^6^ N m^−2^) which means hard to stretch. However, by the geometric construction of the spiral steel wire, it can improve the stretchability of the electrode. Because the tensile properties of silicone rubber are much better than the spiral steel wire, the stretching degree of the entire device is mainly determined by the tensile properties of the spiral steel wire. From the geometrical diagram of the spiral steel wire (Fig. 3a), the relationship between the geometric dimensions is:

1 $$\alpha = \tan^{ - 1} \left( {\frac{t}{{\pi D_{2} }}} \right)$$

2 $$D = D_{2} + d$$

where *D* is outer diameter, *D*_2_ is medium diameter, *t* is pitch, *α* is spiral angle and *d* is diameter of steel wire.

In the linear elastic range of the spiral steel wire, the uniaxial tensile deformation is proportional to the external force, following Hooke’s law [40]:

3 $$H_{\text{x}} - H_{0} = \lambda = \frac{{F_{\text{x}} }}{k}$$

where *H*_0_ is the initial length, *H*_x_ is the length of the spiral steel wire after being enlarged, *λ* is the deformation, *F*_x_ is the tensile force and *k* is elastic modulus. When strain degree of the spiral steel wire reached 51.9%, it reaches the elastic limit, which means the recoverable stretch reaching the maximum (Fig. S4). Therefore, for a single device, it exhibits good stretchability, which can be enlarged as much as 53.4% (Fig. 3b). Meanwhile, the elastic mold (*k*) of FTS-TENG (2.18 N cm^−1^) is close to that of spiral steel wire (1.94 N cm^−1^) in the stress–strain curves. Here, the degree of enlargement H is defined as:

4 $$H = \frac{{H_{\text{x}} - H_{0} }}{{H_{0} }} \times 100\% = \frac{\lambda }{{H_{0} }} \times 100\% .$$

As indicated by the photograph of the FST–TENG at original state (Fig. 3c) and stretched state (Fig. 3d), the diameter of the FST–TENG reduces from ~ 2.978 mm at original state to ~ 2.01 mm at 50% strain, while the resistance of the steel wire electrode was almost unchanged (Fig. 3e). The output signals under the different stretched degree of FST–TENG were acquired, as shown in Fig. 3f–h. With the elongation of the FST–TENG, *V*_oc_, *I*_sc_, and *Q*_sc_ initially increase and recover at same value. The contact area between skin and the FST–TENG remains unchanged as shown in Fig. S5. Meanwhile, the positive effect of the thinner silicone rubber (small effective distance, *d*_0_) during stretching affects the output, which can be explained by the Poisson’s effect [20, 41, 42]. Thus, the output increases fast from 0% to 30% strain and then the output increases slightly from 30 to 50%.

**Fig. 4:**
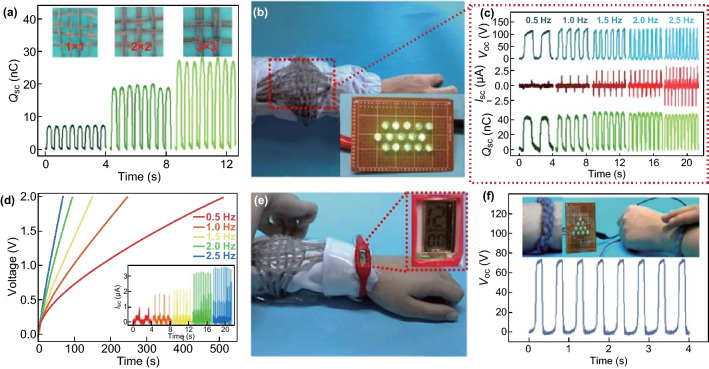
Demonstration of the FST–TENG-based fabric as wearable power source. **a** *Q*_sc_ of the TENG fabric with knitting patterns of 1 × 1, 2 × 2, and 3 × 3 nets. **b** Photograph of the FST–TENG-based fabric, which enables to light up 15 green LEDs by hand tapping. **c** Electrical outputs of the TENG fabric under various motion frequencies ranging from 0.5 to 2.5 Hz, including *V*_oc_, *I*_sc_, and *Q*_sc_. **d** Charging curves of a capacitor (10 μF) charged by the FST–TENG fabric at various motion frequencies. Inset shows the current output of the FST–TENG fabric after rectification. **e** Photograph of the self-charging powered system driving an electronic watch. **f** *V*_oc_ of the FST–TENG-based bracelet at the motion frequency of 2 Hz. Inset shows the photograph of 10 green LEDs lighted up by hand tapping

To make the FST–TENG suitable for wearing on the human body, a FST–TENG based fabric was fabricated as self-charging wearable power source, as demonstrated in Fig. 4. In order to connect several FST–TENGs in parallel that knitted into cloth, three knitting patterns of 1 × 1, 2 × 2, and 3 × 3 type of FST–TENG fabric were woven. The *Q*_sc_ of the fabric increases with an increasing number of single devices due to the increase contact area (Fig. 4a). Meanwhile, the *I*_sc_ follows the same tendency (Fig. S6). When manually tapped 12 fibers with 1 × 1 knitting, the FST–TENG fabric can sufficiency light up 15 LEDs in series (Fig. 4b and Movie S1). To systematically investigate the output performance, the as-fabricated FST–TENG fabric was evaluated at contact-separation motion in single-electrode mode. With the increase of the working frequency from 0.5 to 2.5 Hz, the *I*_sc_ rises from 0.87 to 3.23 μA, while both the *V*_oc_ and *Q*_sc_ do not changes and stay at ~ 121.8 V and ~ 45.8 nC, respectively (Fig. 4c). However, by increasing load resistance, the average output power of the FST–TENG fabric reaches the maximum value of 4.06 μW at an external load resistance of 100 MΩ (Fig. S7). Due to the pulsed and alternating output characteristic, the generated electricity of FST–TENG needs to be converted by the bridge rectifier from alternating to direct current and then stored into the storage devices. Figure S8 shows the working circuit of the self-charging powered system. After rectifying, the current output of FST–TENG remains almost same with the value before rectifying (inset of Fig. 4d). The voltages of the commercial capacitors of 10 μF charged by FST–TENG fabric under different working frequencies were measured to evaluate the charging capacity. It takes ~ 68 s to charge the commercial capacitor to 2 V at 2.5 Hz. When tapping the FST–TENG fabric with hand, it takes ~ 40 s to charge commercial capacitor to drive the electronic watch (Fig. 4e and Movie S2). Likewise, FST–TENG can also be knitted into bracelet worn on the wrist to harvest energy. By hand tapping the FST–TENG bracelet, which is knitted by three FST–TENGs, the *V*_oc_, *I*_sc_, and *Q*_sc_ can reach ~ 73 V, ~ 1.12 μA, and ~ 26 nC, respectively (Figs. 4f and S9). Moreover, the FST–TENG bracelet enables to light up 10 LEDs in series (inset of Fig. 4f).

**Fig. 5:**
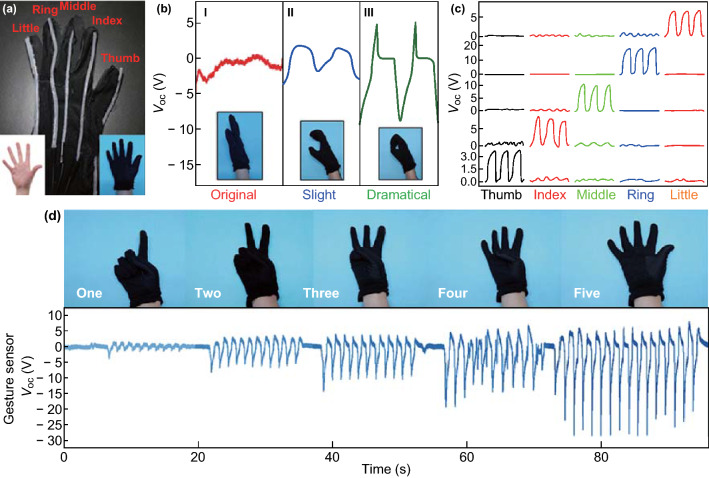
Demonstration of the FST–TENGs woven in a smart glove as active gesture sensor. **a** Photograph of the smart glove with FST–TENGs woven on dorsum and demonstration of the operation mode. **b** Photograph and corresponding *V*_oc_ of the smart glove under different degree of finger bending, including original state, slightly and dramatically bending. **c** *V*_oc_ signals of the five fingers by buckling from the thumb to the little finger in turn. **d** Demonstrations of the smart glove for representing the numbers of “one”, “two”, “three”, “four” and “five” by different gestures

Besides, the FST–TENG can be applied as active physiological motion and human–machine interface sensor due to its high sensitivity and rapid response/recovery time for low-frequency movements [43–46]. Here, it can be embodied in the gesture sensing when constructed into a smart glove, as demonstrated in Fig. 5. Figure 5a shows that the FST–TENGs are parallel connected and sew on the back of the glove corresponding to the position of five fingers. When any finger is bent and released, the contact area between the skin and FST–TENG increases and decreases at the same time. Thus, the real-time voltage could indicate the different gestures of testers, as shown in Fig. 5b. It is remarkable that the fingers have very weak jitter when they are not bent, but it does not affect as the contrast (state I). Compared with slightly bending, the dramatically bending results in the larger contact area, which would induce larger change in the electric potential of the steel electrode. Therefore, the voltage output of the five fingers collected by bending in large angle was much higher than that in small angle (state II and III and Movie S1). To adapt to different frequencies of human motion, it was tested that the same voltage value can be maintained at different frequencies (Fig. S10), due to the constant contact area of the same bending degree. When connecting these 5 FTS-TENG devices to 5 signal acquisition terminals separately, the voltage signals of the five fingers are collected by bending them in turn from the thumb to the little finger and transmitted to the computer, as shown in Fig. 5c. There is a phenomenon in the human finger that when a finger is bent, other fingers will also have slight resonant reactions. However, the *V*_oc_ of the finger at the bending state are significantly higher than those at the immobile states, indicating that the smart glove can effectively identify real finger in bending. In addition, it is also obvious that the electrical outputs of this smart glove increased with the number of bent fingers increases from one (~ 3 V) to two (~ 9 V), three (~ 14 V), four (~ 20 V), and five (~ 32 V) (Fig. 5d and Movie S2), further verifying its feasibility for use in recognition of different gestures.

## Conclusions

In summary, a spiral steel wire-based TENG has been proposed for harvesting human motion energy and inspecting gesture. A single FST–TENG with the length of 6 cm and diameter of ~ 3 mm worked in the single-electrode mode at 2.5 Hz enables to generate *V*_oc_ of ~ 59.7 V, *Q*_sc_ of ~ 23.7 nC, maximum *I*_sc_ of ~ 2.67 μA and average power of ~ 2.13 μW, respectively. Moreover, the FST–TENG can be used even after been twisted, bended and tailored into designed shape for enhancing user experience without impairing its performance. After knitting the FST–TENGs to an energy-harvesting FST–TENG fabric worn on the human body or a bracelet worn on wrist, the FST–TENG fabric with 1 × 1 knitting 12 fibers were demonstrated to light up at least 15 LEDs and charge a commercial capacitor to 2 V in ~ 68 s, and then drive an electronic watch. Meanwhile, the FST–TENG bracelet can light up 10 LEDs in series by hand tapping as well. With these excellent performances, the highly stretchable FST–TENG fabrics can be used as a wearable and convenient power source to harvest human motion energy for wearable electronics. Besides that, the FST–TENG can be woven on dorsum of glove to monitor the movements of gesture, which can recognize every single finger, different bending angle and numbers of bent finger by the voltage signals.

## Electronic supplementary material

Below is the link to the electronic supplementary material.

Supplementary material 1 (PDF 635 kb)

Supplementary material 2 (AVI 611 kb)

Supplementary material 3 (AVI 1135 kb)

Supplementary material 4 (AVI 1479 kb)

Supplementary material 5 (AVI 2855 kb)
