# Supplementary material for: Spiral Steel Wire Based Fiber-Shaped Stretchable and Tailorable Triboelectric Nanogenerator for Wearable Power Source and Active Gesture Sensor
Source: Nanomicro Lett. 2019 May 11;11:39. doi: 10.1007/s40820-019-0271-3 (PMC7770917; doi:10.1007/s40820-019-0271-3)
Supplement: Supplementary file 1 — Supplementary material 1 (PDF 635 kb) [file 40820_2019_271_MOESM1_ESM.pdf]

Supporting Information for

Spiral Steel Wire Based Fiber-shaped Stretchable and Tailorable Triboelectric Nanogenerator for Wearable Power Source and Active Gesture Sensor

Lingjie Xie[1, #], Xiaoping Chen[1, #], Zhen Wen[1, 2, *], Yanqin Yang[1], Jihong Shi[1], Chen Chen[1], Mingfa Peng[1], Yina Liu[3], Xuhui Sun[1, *]

[1]Institute of Functional Nano and Soft Materials (FUNSOM), Jiangsu Key Laboratory for Carbon-Based Functional Materials and Devices, Soochow University, Suzhou 215123, People's Republic of China

[2]Nantong Textile & Silk Industrial Technology Research Institute, Jiangsu Industrial Technology Research Institute of Textile & Silk, Nantong 226314, People's Republic of China

[3]Department of Mathematical Sciences, Xi'an Jiaotong-Liverpool University, Suzhou 215123, People's Republic of China

[#]L. Xie and X. Chen contributed equally to this work

*Corresponding authors. E-mail: wenzhen2011@suda.edu.cn (Z. Wen); xhsun@suda.edu.cn (X. Sun)

Supplementary Figures

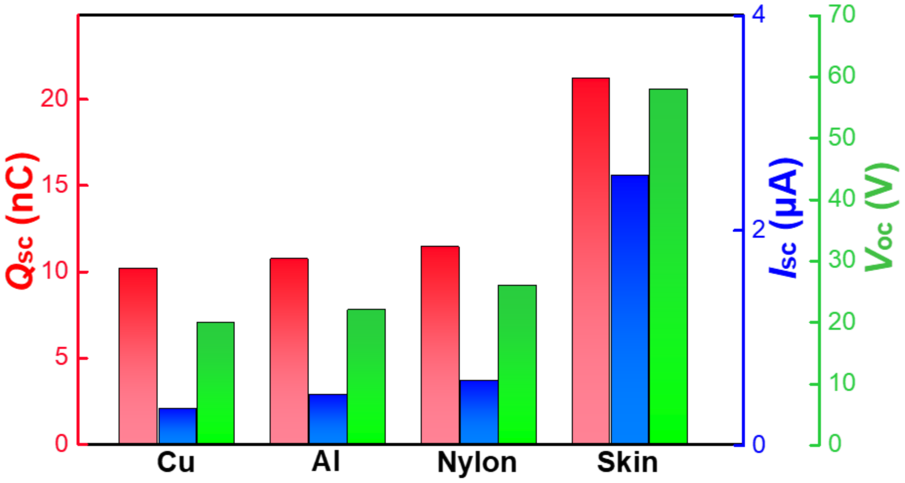

Fig. S1 Short-circuit transferred charge (Q_{sc}), open-circuit voltage (V_{oc}) and short-circuit current (I_{sc}) of the spring based TENG contacting with different materials at 2.0 Hz

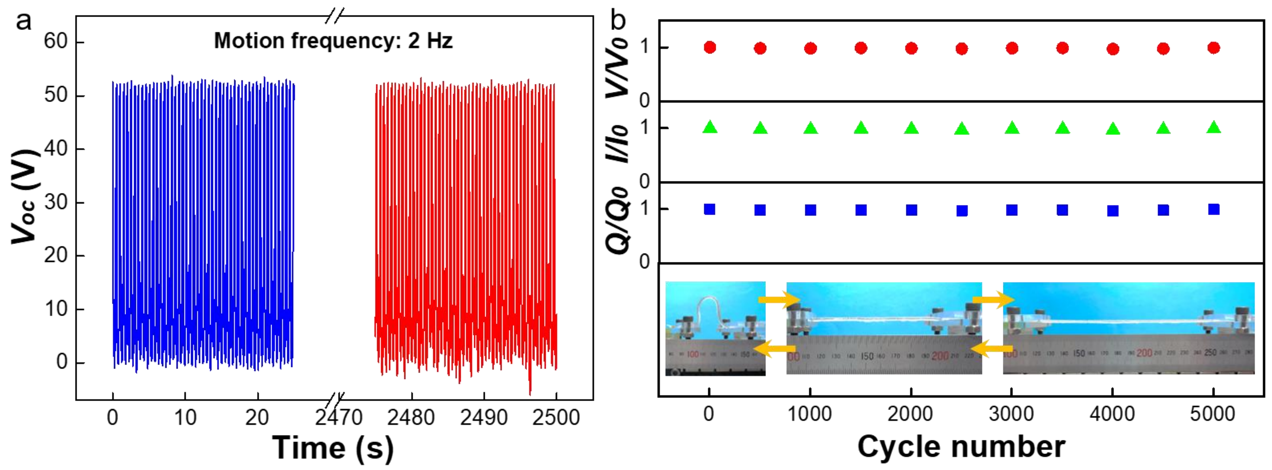

Fig. S2 a V_{oc} of the FST-TENG cycling for 5000 cycles at the working frequency of 2 Hz. **b** Normalized V_{oc}, I_{sc}, and Q_{sc} values of FST-TENG between 180° bended and 50% strain for 5000 cycles. Insets show the photographs of the three status cyclically, including 180° bended, 0° bended and 50% strain

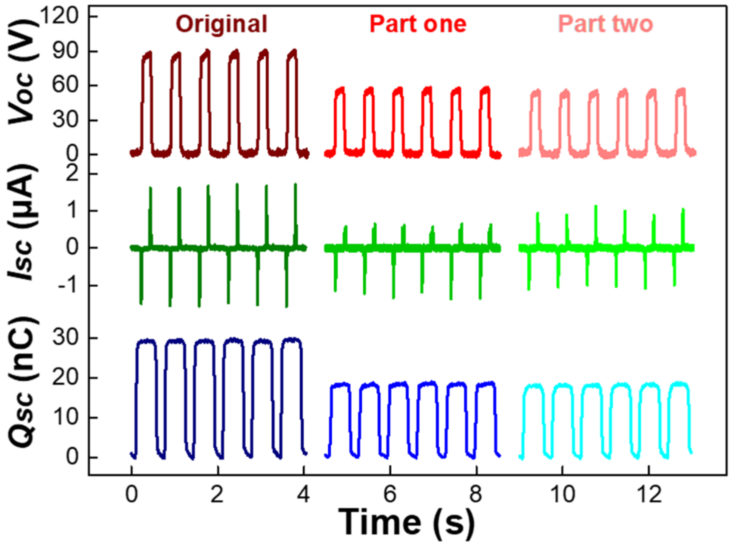

Fig. S3 Demonstration of the tailorability of the FST-TENG

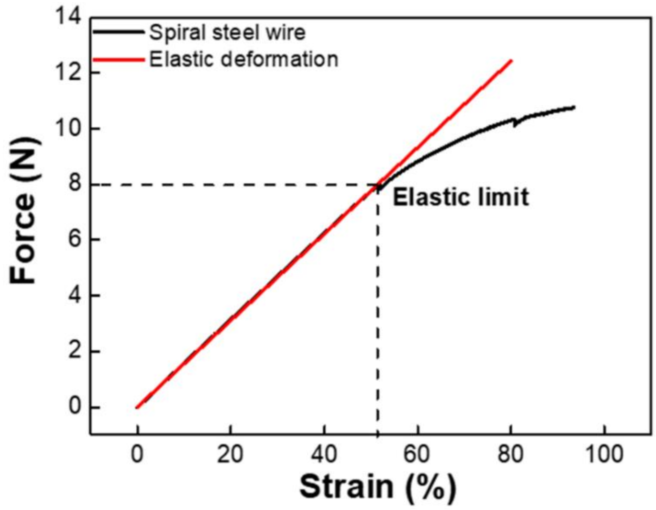

Fig. S4 Stress-strain curve of the spiral steel wire spring

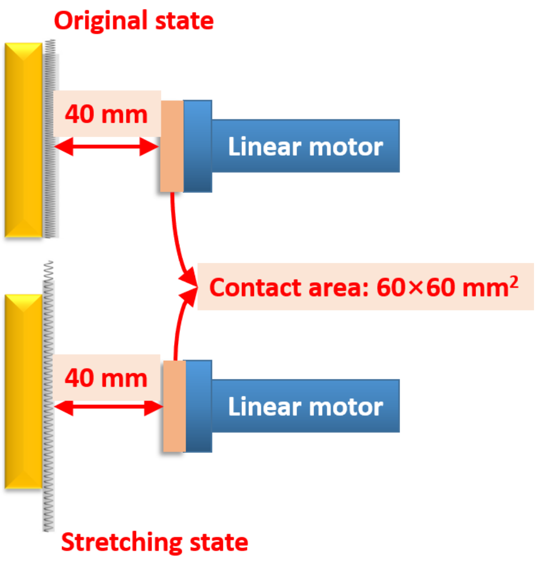

Fig. S5 Schematic diagram of the experimental test method at original and stretching state

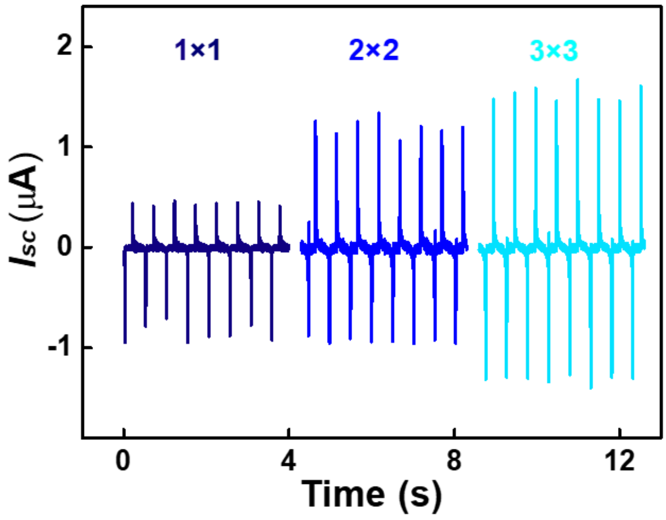

Fig. S6 I_{sc} of the SF-TENG fabric with knitting patterns of 1×1, 2×2, and 3×3 nets

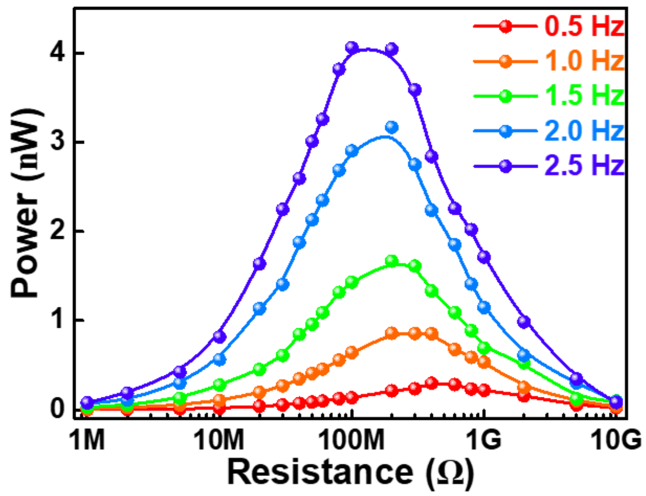

Fig. S7 Dependence of the output power of the SF-TENG fabric under various external load at motion frequencies ranging from 0.5 to 2.5 Hz

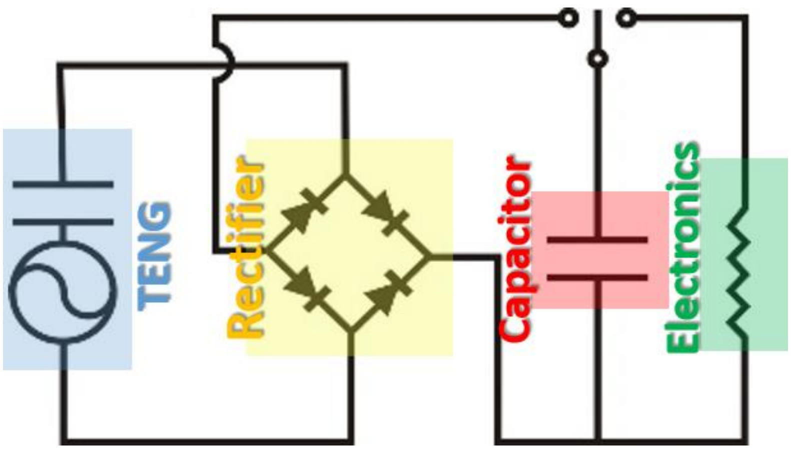

Fig. S8 Equivalent circuit of a self-charging power system based on the SF-TENG fabric as energy harvester for powering electronics

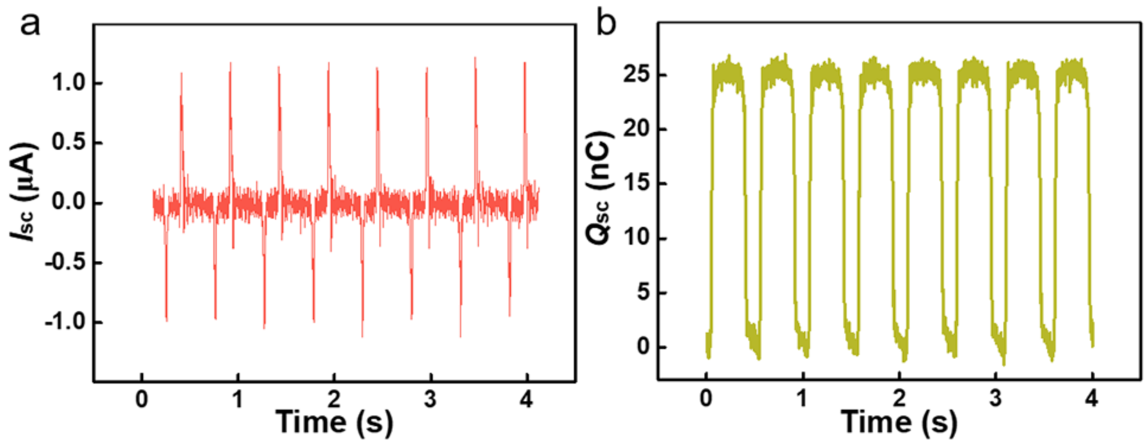

Fig. S9 **a** I_{sc} and **b** Q_{sc} of the SF-TENG bracelet at the motion frequency of 2 Hz

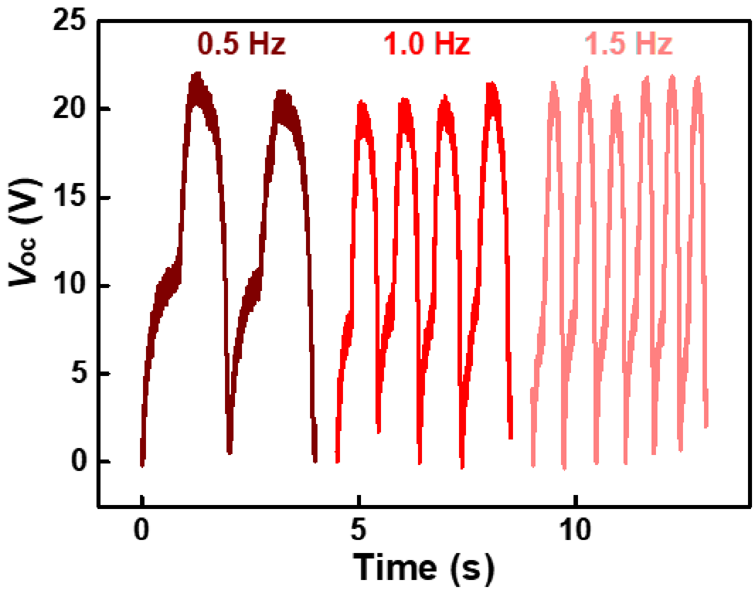

Fig. S10 Open-circuit voltage (V_{oc}) of the smart glove under different frequencies on the same degree of finger bending
